# Surgery and adjuvant radiotherapy *vs* concurrent chemoradiotherapy in stage III/IV nonmetastatic squamous cell head and neck cancer: a randomised comparison

**DOI:** 10.1038/sj.bjc.6602696

**Published:** 2005-07-12

**Authors:** K-C Soo, E-H Tan, J Wee, D Lim, B-C Tai, M-L Khoo, C Goh, S-S Leong, T Tan, K-W Fong, P Lu, A See, D Machin

**Affiliations:** 1 Department of Surgical Oncology, National Cancer Centre, Singapore; 2 Department of Medical Oncology, National Cancer Centre, Singapore; 3 Department of Therapeutic Radiology, National Cancer Centre, Singapore; 4 Centre for Molecular Epidemiology, Occupational and Family Medicine, National University of Singapore, Singapore; 5 Department of Community, Occupational and Family Medicine, National University of Singapore, Singapore; 6 Clinical Trials & Epidemiology Research Unit, Singapore; 7 Department of Otolaryngology, Tan Tock Seng Hospital, Singapore; 8 Department of Otolaryngology, Singapore General Hospital, Singapore; 9 Department of Otolaryngology, Changi General Hospital, Singapore; 10 Department of Surgery, Changi General Hospital, Singapore

**Keywords:** randomised, squamous cell head and neck cancer, chemotherapy

## Abstract

We compared concurrent combination chemotherapy and radiotherapy with surgery and adjuvant radiotherapy in patients with stage III/IV nonmetastatic squamous cell head and neck cancer. Patients with non-nasopharyngeal and nonsalivary resectable squamous cell head and neck cancer were randomised to receive either surgery followed by adjuvant radiotherapy (60 Gy over 30 fractions) or concurrent combination chemotherapy and radiotherapy (66 Gy in 33 fractions). Combination chemotherapy comprised two cycles of i.v. cisplatin 20 mg m^− 2^ day^− 1^ and i.v. 5-fluorouracil 1000 mg m^− 2^ day^− 1^, both to run over 96 h given on days 1 and 28 of the radiotherapy. A total of 119 patients were randomised. At a median follow-up of 6 years, there was no significant difference in the 3-year disease-free survival rate between the surgery and concurrent chemoradiotherapy (50 *vs* 40% respectively). The overall organ preservation rate or avoidance of surgery to primary site was 45%. Those with laryngeal/hypopharyngeal disease subsite had a higher organ-preservation rate than the rest (68 *vs* 30%). Combination chemotherapy and concurrent irradiation with salvage surgery was not superior to conventional surgery and postoperative radiotherapy for resectable advanced squamous cell head and neck cancer. However, this form of treatment schedule with a view to organ-preservation can be attempted especially for those with laryngeal/hypopharyngeal and possibly oropharyngeal disease subsites.

The majority of the patients with squamous cell head and neck cancer (SCHNC) present with locally and/or regionally advanced disease and the use of radical surgery and/or radiotherapy in this setting yield low locoregional control rates and 5-year survival rates not exceeding 40%.

The administration of chemotherapy and radiotherapy concurrently makes use of the resultant synergistic activity to improve tumour cell kill. This strategy has found success in anal canal carcinoma, allowing high cure rates while obviating the need for radical surgery. Studies carried out in the 1990s using combination chemotherapy with concurrent radiation in SCHNC have shown this treatment approach to be feasible despite the significantly higher toxicity and have produced encouraging results. Adelstein *et al* in a phase II trial using cisplatin (CDDP) and 5-fluorouracil (5FU) combination with concurrent split-course radiotherapy have reported a 4-year relapse-free survival of 45% and an overall survival of 49% (Adelstein *et al*, 1993). This when compared retrospectively with a similar patient population treated with radiation alone was shown to be improved. Adelstein next investigated the use of the same combination regimen concurrently with a continuous course of radiotherapy (Adelstein *et al*, 1994). In 19 patients treated in this fashion, despite significant toxicity, there were no treatment-related deaths. At a median follow-up of 20 months, the projected Kaplan–Meier estimate of locoregional disease control was 92%, with the projected relapse-free survival of 86%. Of significance was that primary-site resection was not required in any patient for tumour control. The same group went on to a phase III randomised study comparing radiation therapy alone *vs* concurrent CDDP/5FU and continuous course radiotherapy in 100 patients with resectable stage III and IV SCHNC (Adelstein *et al*, 1997). Salvage surgery was planned for patients whose disease was not responding or progressing when re-evaluated at 55 Grays (Gy). At a median follow-up of 3 years, the 3-year projections of relapse-free survival rates were 52% for patients who received radiotherapy alone and 67% for those who received concurrent chemoradiotherapy. Despite the significant decline in incidence of systemic failure in those who received chemoradiotherapy, the overall survival was not significantly different between the two groups. However, if overall survival with successful primary site preservation was considered, there was significant benefit in favour of those who received chemoradiotherapy (35% with radiotherapy alone *vs* 57% with chemoradiotherapy).

To date, no group has undertaken a randomised trial to compare surgery and adjuvant radiation with concurrent chemoradiotherapy in patients with resectable locally advanced SCHNC. While it is felt that radical radiotherapy with surgery reserved for salvage purposes is equivalent in efficacy to surgery followed by radiation, there has been no randomised trial to support this. Hence, it is still not established if concurrent chemoradiotherapy as used in Adelstein *et al*'s study is superior, or if not, at least equivalent to surgery upfront followed by adjuvant radiation.

Hence, this study was designed primarily to compare the efficacy of concurrent use of CDDP/5FU and radical radiotherapy with surgery upfront followed by adjuvant radiotherapy in patients with resectable nonmetastatic stage III/IV SCHNC.

## PATIENTS AND METHODS

### Eligibility criteria

Patients with newly diagnosed, histologically proven, resectable nonmetastatic stage III/IV SCHNC (excluding nasopharynx and salivary glands) were eligible. In addition, they need to have good performance status of ECOG (Eastern Cooperative Oncology Group) 0 or 1, and adequate bone marrow, renal and hepatic function in order to withstand the rigors of concurrent chemoradiotherapy. Computed tomography scans, CXR and a triple endoscopy were requisite staging procedures prior to recruitment. An informed written consent is required and the protocol was approved by the respective ethics committee of the participating institutions.

### Randomisation and treatment

Patients recruited were randomised to either of the two treatment arms: the standard arm (S) consisted of radical surgery followed by adjuvant radiotherapy and the experimental arm (C) consisted of combination chemotherapy (CDDP/5FU) administered concurrently with radical radiotherapy. Stratified randomisation was carried out using the minimisation method based on the following factors: primary site (oral cavity/oropharynx *vs* larynx/hypopharynx *vs* others) and nodal status (node negative *vs* node positive).

Surgery included a wide resection of the tumour with comprehensive neck dissection for unilateral or bilateral disease as needed. Comprehensive neck dissection for node-positive disease involved the removal of levels I–V lymph nodes. Prophylactic neck dissection was carried out for selected N0 disease. Frozen section controlled-margin was used to ensure clear margins during the surgical procedure. Adjuvant radiotherapy was given to the primary tumour and upper neck at 2 Gy per fraction, 5 days a week to a total of 60 Gy in 30 fractions in 6 weeks. Treatment would commence as soon as adequate healing has been established and not later than 6 weeks after surgery. Fields were reduced to exclude the spinal cord at 40 Gy and a posterior electron-matching field was applied. The dose to clinically uninvolved nodal region was 50 Gy. In patients with disease extending low down the neck, an anterior based AP/PA field was treated to a dose of 50 Gy, to be followed by lateral fields to another 10 Gy, which did not include the spinal cord in the treatment volume. In patients with positive surgical margins, the dose to the area at risk was brought up to 70 Gy using reduced volumes. The lower neck was treated if there was nodal disease present in the upper neck. This lower anterior neck was treated at 2 Gy per fraction to a total dose of 50 Gy in 25 fractions in 5 weeks.

Patients randomised to arm C would receive two cycles of chemotherapy comprising CDDP at 20 mg m^− 2^ day^− 1^ and 5FU at 1000 mg m^− 2^ day^− 1^, both given as a continuous intravenous infusion for 96 h on days 1 and 28 of the radiotherapy course. Radiotherapy in this arm was identical to arm S with the following exceptions: the total dose to the primary tumour and upper neck was 66 Gy in 33 fractions given in six and a half weeks and involved nodes received at least 60 Gy. Patients with positive nodal disease at the outset would undergo elective neck dissection 4–6 weeks postchemoradiotherapy regardless of response.

### Follow-up and salvage surgery

Upon completion of the allocated treatment, the patients were followed up monthly for the first year, two monthly for the second year, three monthly for the third year and 6 monthly thereafter. Patients treated on the C arm underwent examination under anaesthesia about 6–8 weeks post-treatment to evaluate response. In the presence of persistent disease at the primary site, salvage surgery would ensue. Those who achieved complete response but who had nodal disease at the outset would undergo elective neck dissections regardless of response.

### Statistical considerations

It was anticipated that the disease-free survival rate at 3 years with surgery and adjuvant radiotherapy would be approximately 50%. Based on the regimen as used by Adelstein *et al* in their study, a 3-year disease-free survival of about 70% can be expected. In order to detect a difference of 20% between the two arms, with a two-sided test size of 5% and power 80%, a recruitment of 200 patients would be required for this study.

### End point definitions

The patient was considered to have an event only if the relapse occurred after the completion of all primary treatment (i.e. surgery and radiation for patients randomised to the S arm and chemoradiotherapy with/without neck dissection or salvage surgery for persistent disease for patients randomised to the C arm). For instance, if a patient treated on the C arm failed to achieve complete response at re-evaluation and the subsequent salvage surgery was complete, then he would be considered disease-free. Overall survival is defined as the time from randomisation to the time when the patient was known to be alive.

## RESULTS

### Patient cohort

**Figure 1:**
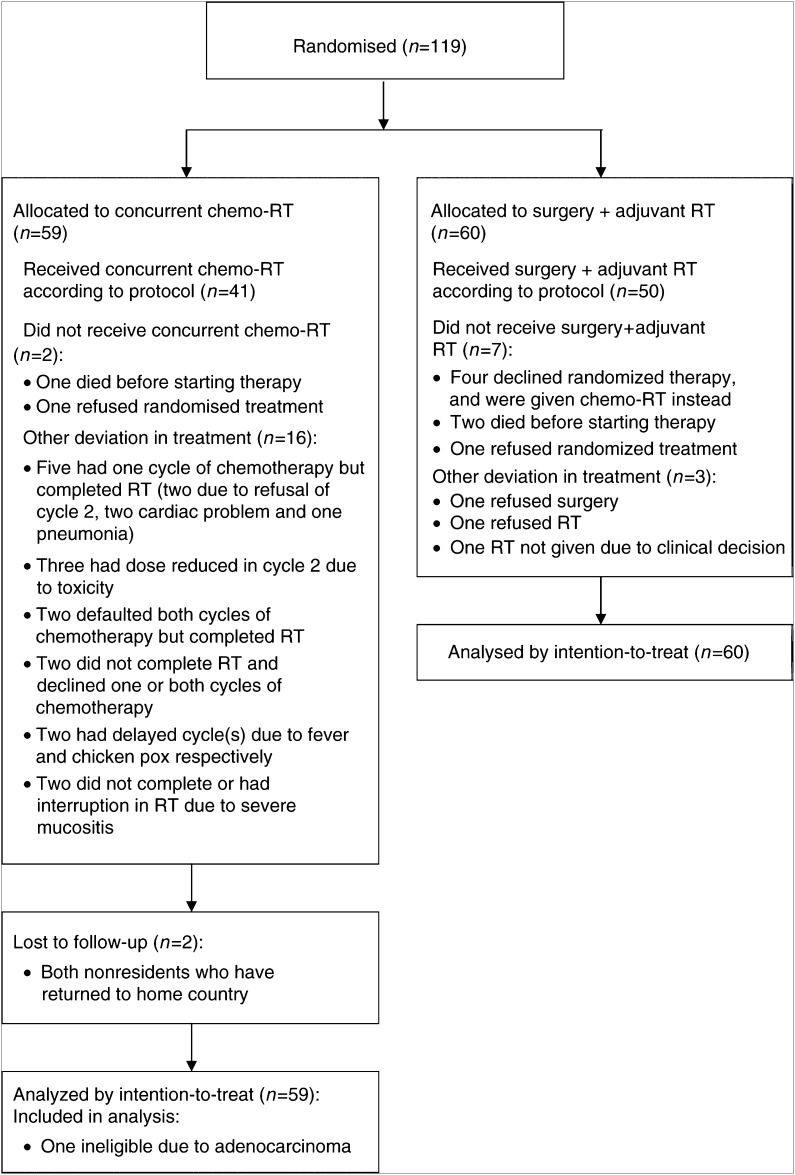
Trial profile.

A total of 119 (59 C, 60 S) patients were randomised between 19 August 1996 and 21 February 2002. Due to the slow accrual rate, the data monitoring committee (DMC) recommended that the accrual to the study should stop as it was deemed unlikely that the target could be reached within a reasonable period. One patient in arm C was ineligible because of confirmed adenocarcinoma by histology (Figure 1). Two patients randomised to arm C were lost to follow-up. Nevertheless, they were included in the analysis for the duration that they were observed. The median follow-up time was 6 years.

Table 1 shows that the patients in both treatment arms were comparable in terms of demographic and clinical characteristics. Their median age was 59 years (range 27–75 years), and well-over four-fifths of them were males. The racial composition was 80% Chinese, 11% Indians and 9% Malays.

Oral cavity (27%), supraglottis (23%) and oropharynx (21%) were the main sites of disease. Patients were staged according to the AJCC/UICC system. The majority of the patients had tumour status of T4 (56%), followed by T3 (26%). In addition, for most of them, the nodal status was N2 (46%) or N0 (30%). The distribution of disease stages was 20% Stage III, 75% Stage IVA and 5% Stage IVB.

### Treatment compliance

In total, 50 patients in arm S were treated according to protocol, receiving both surgery and the recommended dose of adjuvant radiotherapy. Four patients who declined the randomised regimen were administered concurrent chemoradiotherapy. Two patients died before starting the randomised therapy, and one refused the allocated treatment. One patient declined only surgery, another only radiotherapy, and yet another was not administered radiotherapy because of clinical decision (Figure 1). Thus, of the 52 patients who had surgery, the resection was complete for 46, grossly complete for five (positive margin on paraffin section), and unresectable for one.

For those randomised to arm C, 41 (70%) received full protocol treatment. The allocated treatment was not administered to two patients as one died before starting therapy, while another refused the randomised option. Other deviations in treatment occurred in 16 patients. Five patients who completed radiotherapy received only one cycle of chemotherapy – two due to refusal of cycle 2, and one each due to acute myocardial infarction, heart problem or pneumonia, respectively. The chemotherapy dose was reduced in cycle 2 for three patients because of toxicity. There were two patients who completed radiotherapy but defaulted both cycles of chemotherapy, while another two did not complete radiotherapy and declined at least one cycle of chemotherapy. Two patients had delayed cycle(s) due to fever or chicken pox, respectively. In addition, one patient each did not complete or had interruption in radiotherapy due to severe mucositis.

In summary, 12% (seven out of 60 patients) of those on arm S and 12% (seven out of 59 patients) of those on arm C failed to comply by deciding against recommended treatment while 5% (three out of 60 patients) of those on arm S and 19% (11 out of 59 patients) on arm C failed to comply due to medical complications.

### Treatment toxicity and surgical complications

The toxicity during treatment was classified based on the RTOG (Radiation Therapy Oncology Group) common toxicity criteria. There were notably higher incidences of toxicity among patients in the C arm. A total of eight patients on arm S and 39 on arm C, experienced toxicity due to radiotherapy and chemoradiotherapy, respectively. For both groups, the most commonly noted Grade 3 toxicity was mucositis (23 C, 5 S), pharyngitis (20 C, 2 S) and moist skin desquamation (18 C, 3 S). Details of other types of toxicity are displayed in Table 2. As noted, all Grade 4 toxicities occurred on the C arm in eight patients. The most common Grade 4 toxicity was neutropenic sepsis, involving five patients. Three patients had Grade 4 neutropenia. There were no toxic deaths from chemoradiotherapy.

For patients randomised to arm S, surgical complications were reported in 14 (27%) patients. One of the complications required lattisimus dorsi pedicled flap, while the rest included spontaneous closure of salivary leak, anastomotic breakdown, superficial neck abscess requiring incision and drainage, complication due to small salivary fistula, lower lid ectropion, aspirated seroma, pharyngeal fistula, C5 root paresis on shoulder, left Horner's syndrome, wound dehiscence, nasal regurgitation when drinking, wound infection at the right neck as well as a complication involving right neck fistulae, bilateral chylothorax and pneumonia. Three of these 14 patients had also experienced Grade 3 toxicity involving moist skin desquamation, mucositis and/or pharyngitis.

### Clinical response to concurrent chemoradiotherapy

An evaluation of all patients on arm C with regard to tumour response was conducted at 6 weeks post-treatment. Only 56 patients recorded information on tumour response as one patient died before treatment, another refused the randomised option, and yet another died before he was evaluated for tumour response. Table 3 shows that 39 patients responded completely to treatment, yielding a complete response rate of 70% (95% CI: 57–80%).

### Salvage surgery and radical neck dissection

**Figure 2:**
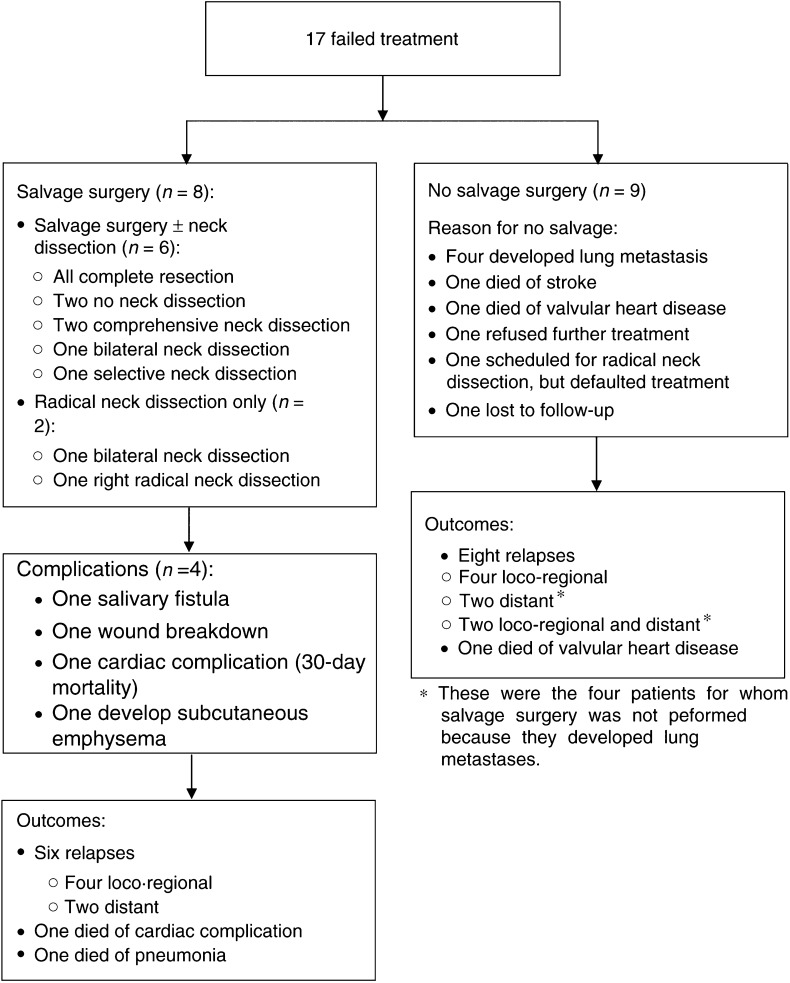
Outcome of patients in C requiring salvage surgery.

Salvage surgery was performed on eight of 17 patients who had or were suspected to have failed chemoradiotherapy (Figure 2). This included one patient for whom salvage surgery was scheduled but only bilateral radical neck dissection was carried out when frozen section of the biopsy from the primary site was negative, and another who had right radical neck dissection only. All of these patients had partial tumour response. For the six patients who had salvage surgery of the primary site, complete resection was attained for all of them. In addition, one each had a bilateral and a selective neck dissection, and two had comprehensive neck dissection. Complications occurred in four patients: salivary fistula, wound breakdown requiring flap reconstruction, a cardiac complication that resulted in death within a week of surgery and development of subcutaneous emphysema due to communication with tracheostomy tube. Four of the eight patients who had undergone salvage surgery and/or radical neck dissection due to failed chemoradiotherapy later experienced local relapse, two developed distant metastases and one died of other causes.

Of the remaining nine patients on arm C who did not respond to treatment, salvage surgery was not carried out because of lung metastasis (four patients), stroke (one patient), valvular heart disease (one patient) and refusal of treatment (three patients). The latter three patients, together with the one who had stroke, developed loco-regional disease. Two patients each had distant relapse, as well as loco-regional and distant relapses.

In accordance to the protocol, patients with nodal disease at diagnosis who attained complete response after chemoradiotherapy were to undergo elective neck dissection. Of the 39 patients with complete response, 11 were of N1 and 17 N2. However, radical neck dissection was performed on 22 (nine N1, 13 N2). There were six cases of complications: wound breakdown requiring flap revision, right carotid blow-out, chyle leak, postoperative pyrexia and thrombophlebitis, pneumonia as well as wound dehiscence. Of the remaining six patients who did not have RND, the respective reasons for five of them were patient refusal (two cases), development of bronchopneumonia, death from heart failure 1 month after completion of chemoradiotherapy, decision of the attending surgeon not to proceed and unknown for one patient.

Salvage surgery was also performed on three of six patients who subsequently developed local relapse after an initial complete response. Of these three, one developed a second local relapse after salvage surgery, while another died within a month following salvage surgery from a tracheo-inominate fistula as a result of surgical complication. Of the three patients who did not undergo salvage surgery, one refused while the other two were deemed unresectable due to extent of local recurrence.

Thus, among a total of 33 patients who had undergone salvage surgery and/or radical neck dissection, 11 complications were reported in all, yielding a surgical complication rate of 33%.

Altogether, 23 of 54 patients on arm C who were evaluable for primary site organ preservation remained free from salvage surgery (Table 4). All of them responded completely to treatment and had no local relapse. The overall rate of avoidance of surgery was 42% (95% CI: 30–56%). This rate appears to vary with the site of disease, with the laryngeal/hypopharyngeal patients reporting the highest avoidance rate of 62% (95% CI: 41–79%).

### Site of relapse

A total of 57 patients (30 C, 27 S) had relapsed after completion of treatment. Table 5 shows the distribution of site of first relapse. Loco-regional relapse was the most common, occurring in 34 (19 C, 15 S) patients. This was followed by distant metastasis, frequently at the lung, which occurred in 20 patients (10 C, 10 S). There were three (1 C, 2 S) cases of relapse involving both loco-regional and distant metastases.

### Disease-free survival (DFS)

**Figure 3:**
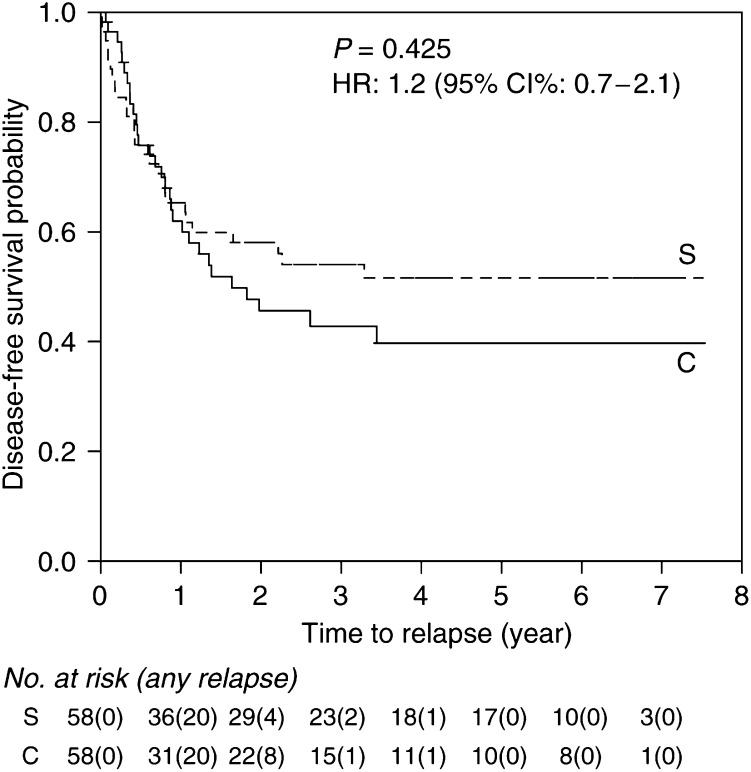
Disease-free survival by treatment. Three patients in S relapsed before completion of radiotherapy. The date of surgery was thus taken as the date of treatment completion. Another patient who died within a week of completion of salvage surgery was considered disease-free.

The Kaplan–Meier DFS curves for the two treatment groups are shown in Figure 3. The median disease-free survival time was 1.6 years for arm C and was not reached for arm S. The 3-year DFS rates for the two groups were 43% for C and 54% for S. This difference was not statistically significant.

### Overall survival (OS)

**Figure 4:**
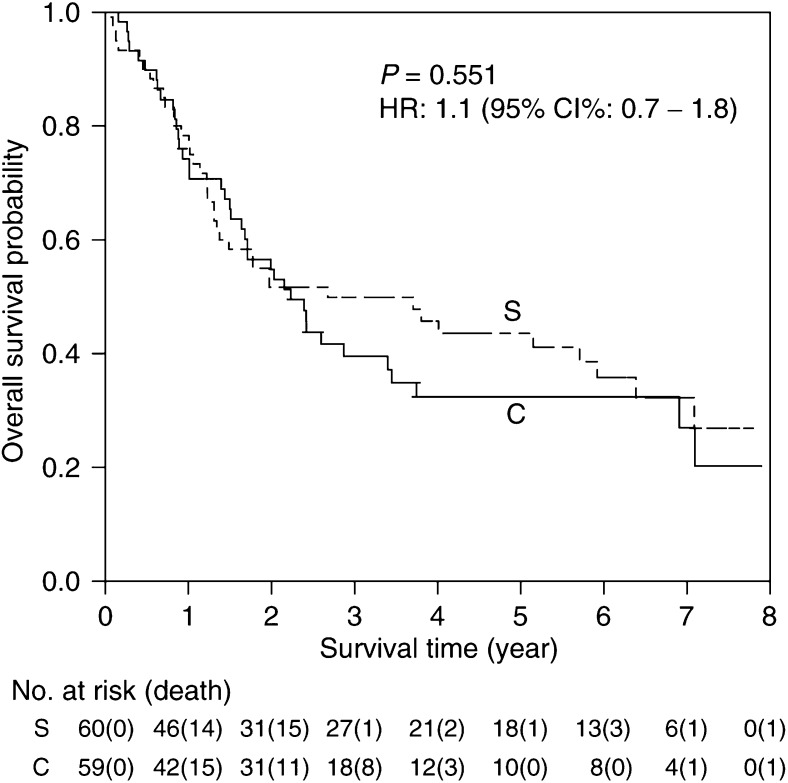
Overall survival by treatment.

At the time of analysis, 77 (39 C, 38 S) patients have died (Table 6). The distribution of cause of death was similar between the two treatment arms. The most common cause was local recurrence, occurring in 27 (13 C, 14 S) patients. There were also 14 (7 C, 7 S) patients who died of both local recurrence and distant metastasis. Of the 27 who died of other causes, five were cardiac-related (2 C, 3 S) and seven were due to pneumonia (4 C, 3 S). Figure 4 compares the Kaplan–Meier OS curves for the two treatment groups. The median overall survival time for arm C was 2.2 years. For those who were randomised to arm S, the median survival time was 2.7 years. The 3-year survival rates for the two groups were 40% for arm C and 50% for arm S. The difference was not statistically significant.

## DISCUSSION

For various reasons, the management of SCHNC poses a significant challenge to the physician and to society as a whole. This condition tends to occur in the socio-economically deprived segment of the population and comorbidities such as cardiovascular disease and chronic obstructive airway disease are frequent in this group due to the pervasive damage from tobacco usage. These two factors can pose significant impediments to radical therapeutic maneuvers and hence eligibility for accrual to clinical trials.

This study, the first attempt ever to compare the upfront surgery and adjuvant radiotherapy with concurrent chemoradiotherapy as primary treatment, faced several problems. The slow accrual rate reflects the unwillingness of patients to have their treatment subject to randomisation. A significant number of patients who were approached for recruitment would prefer to make the choice themselves when given two treatment alternatives.

Noncompliance with allocated treatment was proportionately high. Noncompliance because of decision by the patients against accepting all or part of the recommended treatment was equal in both arms. However, medical complications interfering wholly or partly with the allocated treatment were significantly more common in those randomised to chemoradiotherapy. This observation underscores the significant toxicities associated with such an intense treatment and this could limit the assessment of the true efficacy of this treatment strategy. The development of severe oropharyngeal mucositis often limits the dose and the number of cytotoxic agents that can be used with concurrent radiation to the head and neck region. The frequent coexisting medical conditions in patients with SCHNC could also have contributed to the high morbidities associated with chemoradiotherapy. Nevertheless, 53 patients (90%) received at least one cycle of the combination chemotherapy regimen concurrent with radiotherapy and no treatment-related deaths were encountered (Figure 1). Adelstein *et al* (1994) administered two cycles of 5FU and CDDP on days 1 and 21 of radiation to his cohort of patients, which is more intense than our study. Yet they were able to achieve 100% compliance rate with the chemotherapy and radiation doses. Although the toxicity was significant with 37% neutropaenic rate, no toxic deaths were encountered. In contrast, our experience with a similar, albeit less intense regimen has been less favourable. Only 69% of our patients who received chemoradiotherapy were able to complete the preplanned chemotherapy and radiation doses. It is difficult to offer a clear explanation for this difference. One possible explanation is that our institution is a public institution that caters to the lower socioeconomic group of patients and compromised socioeconomic support may have an adverse impact on treatment compliance. Another possible reason is the ethnic difference between the two groups. However, there is currently scarce data to support ethnic differences in handling the cytotoxics used in the two studies.

Another important observation from our study is the rather limited ability of surgery to salvage recurrence or persistent disease successfully (see Figure 2). The high complication rate plus the high relapse rate after salvage surgery contributes to this high failure rate. Again, our experience with salvage surgery in patients who received chemoradiotherapy as primary treatment differs significantly from that of Adelstein's group (Adelstein *et al*, 2000). Among the 17 patients who failed concurrent chemoradiotherapy in our study, salvage was possible in only 47% of these patients. There were no long-term disease-free survivors in this group of patients (see breakdown in Figure 2). In contrast, salvage surgery was successful in at least local control in eight out of 11 patients (73%) who received chemoradiotherapy in Adelstein *et al*'s study. This difference can be explained at least in part by the fact that the two studies are not comparable in the type of patients selected. Firsty, oropharyngeal, hypopharyngeal and laryngeal subsites comprised the vast majority (96%) of the patients in Adelstein *et al*'s study compared to 56% in our study. Secondly, those with T4 lesions made up only 33% of the patients in Adelstein *et al*'s study compared to 56% in our study. These differences in patient factors and the inability to deliver the planned doses of cytotoxics could have resulted in a significantly poorer outcome of our patients with a 3-year overall survival rate of 40% when compared to 60% in Adelstein *et al*'s study (Adelstein *et al*, 1997).

Our study failed to show an advantage with the use of aggressive combination chemotherapy with concurrent radiotherapy although it is difficult to conclude firmly on this because the accrual target was not reached. As stated above, the patients in our study were mainly those with bulky disease. Hence, it is conceivable that chemoradiotherapy is less likely to lead to complete pathological response in such bulky disease. Radical surgery upfront could be a better approach.

Two studies were published back-to-back recently addressing the role of adjuvant chemoradiotherapy (Bernier *et al*, 2004; Cooper *et al*, 2004). The US RTOG/Intergroup study and EORTC study have similar study design where a randomised comparison was made between the standard adjuvant radiotherapy and the adjuvant CDDP with concurrent radiotherapy. Both studies are also similar in certain outcomes, namely a significantly improved disease-free or progression-free survival and significantly increased treatment-related toxicities both in the combined modality arms. The US study reported 2% treatment-related death rate in the combined arm (Cooper *et al*, 2004). Despite the smaller sample size, the EORTC study was able to show a significant difference in overall survival rate at 5 years (53 *vs* 40%) in favour of the combined arm while the US study could not. This divergence may appear puzzling, but the difference in the composition of the patient population in both studies could account for this. The proportion of patients with laryngeal/hypopharyngeal cancers was clearly lower in the US study (127 out of 416 or 30%) compared with the EORTC study (143 out of 334 or 43%). The EORTC study does suggest that the incorporation of all three modalities may provide an advantage as dual modalities regardless of the scheduling or modalities used have not been shown conclusively to result in improved overall survival outcome in most randomised studies conducted to date. This makes sense, as locally advanced SCHNC is largely a locoregional problem that requires aggressive locoregional therapy to tackle effectively.

Should we consider organ preservation at all for the patients with the appropriate subsites (i.e. larynx or hypopharynx) with bulky disease? This goal is attainable based on the results of our study. However, attempting to preserve organ in patients with bulky disease should be carried out with proper patient selection and close follow-up. Our data suggest that patients with disease involving the laryngeal/hypopharyngeal subsites were more likely to achieve this goal. Another potential candidate is the oropharyngeal subsite with organ preservation successful in 55% of such patients.

A main criticism with the design of this study is the heterogeneity of the disease subsites included. Although confining the study population to one or two disease subsites is ideal and would increase the robustness of the results, we felt that doing so would curtail the accrual rate significantly. Moreover, based on the promising results shown by Adelstein *et al* in their studies, the primary objective of this study was to compare the efficacy of concurrent chemoradiotherapy as primary treatment with upfront surgery followed by adjuvant radiotherapy in patients with locally advanced resectable disease and not organ preservation. However, even the inclusion of heterogeneous subsites did not help us attain the accrual target over the 5-year study period. It is unlikely that a study of similar design can be replicated.

In conclusion, surgery remains an important modality in the management of patients with locally advanced SCHNC, especially those with bulky, yet resectable disease. Concurrent chemoradiotherapy is an effective form of treatment schedule but is limited by its significant toxicities especially in patients who are often compromised by other comorbidities. Experience and availability of good nursing and paramedical supports are necessary requisites to carry out this treatment schedule safely.

## Figures and Tables

**Table 1:** Patient characteristics by treatment

	**C (*n*=59)**	**S (*n*=60)**	**All patients (*n*=119)**
*Age (years)*			
Median	60	58	59
Range	35–73	27–75	27–75
			
*Sex (%)*			
Male	51 (86)	53 (88)	104 (87)
Female	8 (14)	7 (12)	15 (13)
			
*Race (%)*			
Chinese	45 (76)	50 (84)	95 (80)
Indian	8 (14)	5 (8)	13 (11)
Malay	6 (10)	5 (8)	11 (9)
			
*Site of disease (%)*			
Oral cavity	19 (32)	13 (22)	32 (27)
Oropharynx	12 (20)	13 (22)	25 (21)
Hypopharynx	7 (12)	7 (12)	14 (12)
Supraglottis	13 (22)	14 (23)	27 (23)
Glottis	5 (9)	5 (8)	10 (8)
Subglottis	0 (0)	1 (1)	1 (1)
Maxillary sinus	3 (5)	7 (12)	10 (8)
			
*Tumour status (%)*			
T1	0 (0)	5 (8)	5 (4)
T2	9 (15)	8 (13)	17 (14)
T3	16 (27)	15 (25)	31 (26)
T4	34 (58)	32 (54)	66 (56)
			
*Nodal status (%)*			
N0	19 (32)	17 (28)	36 (30)
N1	15 (25)	7 (12)	22 (19)
N2	24 (41)	31 (52)	55 (46)
N3	1 (2)	5 (8)	6 (5)
			
*TNM staging (%)*			
Stage III	12 (20)	12 (20)	24 (20)
Stage IVA	46 (78)	43 (72)	89 (75)
Stage IVB	1 (2)	5 (8)	6 (5)

**Table 2:** Grades 3 or 4 toxicity by treatment

	**C**		**S**	
**Types of toxicity**	**Grade 3**	**Grade 4**	**Grade 3**	**Grade 4**
Mucositis	23	0	5	0
Pharyngitis	20	0	2	0
Moist skin desquamation	18	1	3	0
Neutropaenia	4	3	0	0
Anorexia	3	0	0	0
Laryngitis	2	0	1	0
Leucopaenia	1	0	0	0
Thrombocytopaenia	1	0	0	0
Neutropaenic sepsis	0	5	0	0
Other^a^	1	1	0	0

a Include Grade 3 pneumonia and Grade 4 Hepatitis B.

**Table 3:** Tumour response for patients on C at 6 weeks post-treatment

**Response at 6 weeks**	***N***	**%**
Complete response	39	69.6
Partial response	13	23.2
Static disease	1	1.8
Progressive disease	3	5.4
		
Total	56	100.0

**Table 4:** Avoidance of salvage surgery at primary site

**Site of disease**	**Estimate of proportion**	**95% CI**
Total	23/54^a^ (43%)	30–56%
		
*Laryngeal*	13/21 (62%)	41–79%
Hypopharynx	3/7	—
Supraglottis	7/11	—
Glottis	3/3	—
		
*Nonlaryngeal*	10/33 (30%)	17–47%
Oral cavity	4/19	—
Oropharynx	6/11	—
Maxillary sinus	0/3	—

a Information not available on five patients – one died before starting therapy, one died before evaluation for response, two had complete response, but one was later lost to follow-up and two died of unknown causes.

**Table 5:** Site of first relapse

**Site**	**C**	**S**	**Total**
Loco-regional	19	15	34
Distant metastasis	10	10	20
Both	1	2	3
			
Total	30	27	57

**Table 6:** Survival status by treatment

	**C (*n*=59)**	**S (*n*=60)**	**Total (*n*=119)**
*Alive*			
Disease-free	18	22	40
With local recurrence	0	0	0
With distant metastasis	0	0	0
With both local and distant recurrences	0	0	0
			
*Dead*			
Of local recurrence	13	14	27
Of distant metastasis	5	4	9
Of both local and distant recurrences	7	7	14
Other cause^a^	14	13	27
Lost to follow-up	2	0	2

a Cause of death was not known for one each in C and S arm.
